# Chronic Methamphetamine Administration Increases Mitochondrial Density and Impairs Mitochondrial Function in the Substantia Nigra Pars Compacta

**DOI:** 10.1111/jnc.70554

**Published:** 2026-09-24

**Authors:** Aishwarya Bhowmik, Jennifer A. Wilking, Duha Alshareef, Colin Campbell, Steven M. Graves

**Affiliations:** ^1^ Department of Pharmacology University of Minnesota Minneapolis Minnesota USA

**Keywords:** methamphetamine, mitochondria, monoamine oxidase, substantia nigra pars compacta

## Abstract

Methamphetamine (meth) is an addictive psychostimulant that induces monoamine oxidase (MAO)‐dependent mitochondrial oxidative stress and MAO‐dependent degeneration of substantia nigra pars compacta (SNc) dopamine neurons in male mice, suggesting that meth‐induced mitochondrial oxidative stress is necessary for degeneration. However, the impact of chronic in vivo meth administration on SNc mitochondria is unclear. We therefore examined the impact of chronic meth administration on the expression of mitochondrial fission 1 (FIS1) and Dynamin‐related protein 1 (DRP1), proteins involved in mitophagy, as well as mitochondrial density and mitochondrial function in the SNc. Male C57BL/6J mice received saline or meth (5 mg/kg; i.p.) for 28 days; chronic meth administration decreased SNc expression of both DRP1 and FIS1 proteins, suggesting impaired mitophagy. Consistent with this, mitochondrial density in SNc dopamine neurons was also increased. Moreover, this increase in mitochondrial density was MAO‐dependent, suggesting that chronic meth‐induced SNc degeneration is associated with an increase in mitochondrial density. To determine whether mitochondria were functional, high‐resolution respirometry assessments were conducted using SNc tissue from mice treated with either chronic meth or saline. Results indicate that chronic in vivo meth administration decreased mitochondrial complex I as well as complex I + II oxidative phosphorylation capacities and coupling efficiencies in the SNc. Presented data suggest that chronic in vivo meth administration results in an accumulation of functionally compromised mitochondria in the SNc, which is associated with and may potentially contribute to chronic meth‐induced neurodegeneration.

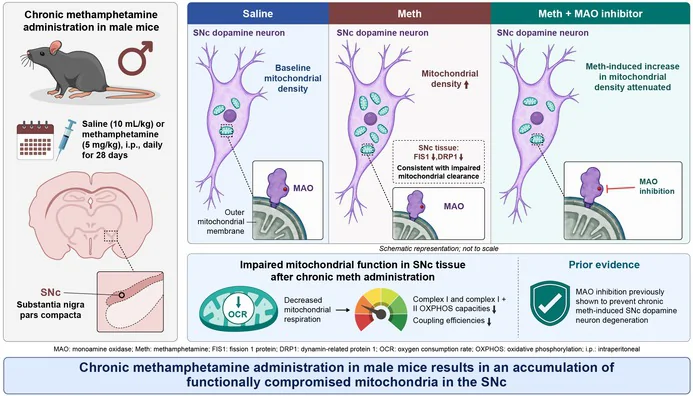

## Graphical abstract

Chronic administration of methamphetamine, an addictive and neurotoxic psychostimulant, results in degeneration of dopamine neurons in the substantia nigra pars compacta of male mice. The current study demonstrates that chronic administration of methamphetamine also results in an accumulation of mitochondria with impaired function in the substantia nigra pars compacta. Prior studies show that inhibition of monoamine oxidase, an enzyme involved in dopamine metabolism, prevents chronic methamphetamine‐induced neurodegeneration of substantia nigra pars compacta dopamine neurons. In the current study, monoamine oxidase inhibition also prevented the observed accumulation of mitochondria. Taken together, evidence suggests that the accumulation of functionally compromised mitochondria may contribute to methamphetamine‐induced neurodegeneration of substantia nigra pars compacta dopamine neurons.

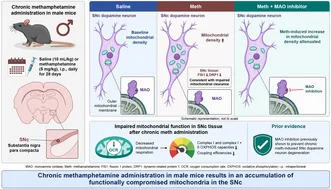

**Abbreviations**
DRP1: dynamin‐related protein 1
FIS1: fission 1 protein
H_2_O_2_
: hydrogen peroxide
MAO: monoamine oxidase
meth: methamphetamine
OCR: oxygen consumption rate
OXPHOS: oxidative phosphorylation
qPCR: quantitative polymerase chain reaction
SNc: substantia nigra pars compacta
TH: tyrosine hydroxylase
VDAC1: voltage‐dependent anion‐selective channel protein 1
VMAT2: vesicular monoamine transporter 2

## 1 Introduction

Methamphetamine (meth) is a potent psychostimulant, and its illicit use continues to be problematic. An estimated 1.8 million people in the United States have a meth use disorder, and of those 1.8 million, over half suffer from a severe meth use disorder (NSDUH 2024). This is particularly alarming given that, in addition to being highly addictive, meth can also cause neurotoxicity, particularly to substantia nigra pars compacta (SNc) dopamine neurons. Clinical studies provide evidence suggesting that meth use can result in a loss of nigrostriatal axons innervating the caudate and putamen (Kitamura et al. 2007; McCann et al. 2008, 1998; Moszczynska et al. 2004; Volkow et al. 2001; Wilson et al. 1996). Additional clinical studies using transcranial sonography suggest that meth‐induced damage to SNc dopamine neurons is not exclusive to axons but may also result in somatic degeneration similar to that seen in Parkinson's disease (Rumpf et al. 2017; Todd et al. 2013; Todd et al. 2016), the most common neurodegenerative movement disorder. Consistent with this, patients with a history of meth abuse are reported to have an increased risk for Parkinson's disease (Callaghan et al. 2010; Callaghan et al. 2012; Curtin et al. 2015; Granado et al. 2013; Lappin and Darke 2021). Using a chronic 28‐day 5 mg/kg meth administration paradigm, we also provide evidence of nigrostriatal axon loss as well as degeneration of SNc dopamine neurons in male mice (Du et al. 2021; Graves et al. 2021; Pilski and Graves 2023).

A primary pharmacological target of meth is the vesicular monoamine transporter 2 (VMAT2) protein; meth binds to and induces dysfunction of VMAT2, resulting in elevated concentrations of cytosolic dopamine (Freyberg et al. 2016; Sulzer et al. 2005). Cytosolic dopamine is then subject to metabolism by monoamine oxidase (MAO) enzymes, which are tethered to the mitochondria (Russell et al. 1979). Dopamine metabolism by mitochondrially tethered MAO results in mitochondrial oxidative stress (Graves et al. 2020). Pretreating mice with either a mitochondrial antioxidant (Du et al. 2021) or an MAO inhibitor (Du et al. 2021; Graves et al. 2021; Pilski and Graves 2023) prevents chronic meth‐induced SNc degeneration. Together, these data suggest that meth‐induced MAO‐dependent mitochondrial oxidative stress is a key driver of SNc degeneration. However, the consequence of chronic in vivo meth administration on mitochondria in the SNc is unclear.

The overall aim of the current study was to determine the impact of chronic in vivo meth administration on mitochondria in the SNc. In vitro, meth decreases cytosolic and mitochondrial levels of dynamin‐related protein 1 (DRP1) and fission 1 (FIS1) (Lenzi et al. 2022). Both of these are proteins involved in mitochondrial fission and mitophagy. We first sought to determine whether similar outcomes resulted in the SNc from chronic in vivo meth administration and, if so, what the functional consequences are. Experimental outcomes from the presented studies provide evidence suggesting that chronic in vivo meth administration impairs the ability to clear mitochondria in the SNc and that this results in an accumulation of functionally compromised mitochondria.

## 2 Materials and Methods

### 2.1 Experimental Subjects and In Vivo Drug Administration

A total of 74 experimental C57BL/6J mice were bred in‐house. All subjects were group‐housed (maximum 4 mice per cage for male mice and 5 mice per cage for female mice) with food and water provided *ad libitum*. Subjects were maintained on a standard 12‐h light/dark cycle, and all experiments were conducted during the light cycle. Mice began receiving daily intraperitoneal injections beginning at approximately 8 weeks of age and throughout the duration of treatment weighed between 18 and 30 g; no randomization was performed to allocate mice in the study. Mice were administered saline (10 mL/kg; General Laboratory Products, Yorkville, IL, USA) or (+)‐methamphetamine hydrochloride (meth, 5 mg/kg; Sigma‐Aldrich, St. Louis, MO, USA), or rasagiline mesylate (1 mg/kg; BioVision, Milpitas, CA, USA) as a 30‐min pretreatment prior to each saline/meth injection for 28 consecutive days. All injections were intraperitoneal and administered in the home cage. Rasagiline is a clinically available MAO‐B inhibitor which attenuates meth‐induced mitochondrial oxidative stress (Du et al. 2021; Graves et al. 2021, 2020) and chronic meth‐induced degeneration of SNc dopamine neurons (Du et al. 2021; Graves et al. 2021). Chronic 28‐day 5 mg/kg meth administration results in a loss of SNc dopaminergic neurons in male (Du et al. 2021; Graves et al. 2021; Pilski and Graves 2023), but not female mice (Pilski and Graves 2023). Similarly, mitochondrial density in female SNc dopamine neurons was unchanged by chronic meth administration (Figure S1). Subsequent experiments in the current report were therefore conducted using male mice. Experimental procedures were in accordance with the Guide for the Care and Use of Laboratory Animals and approved by the University of Minnesota Institutional Animal Care and Use Committee (IACUC Protocol ID 2507‐43133A); mice were monitored twice daily, with routine health checks performed. No adverse reactions were observed, and no mice died prior to experimental endpoints.

### 2.2 Immunoblotting

One day after chronic saline or meth administration, mice (*n* = 5 per treatment group) were anesthetized with isoflurane (up to 5% inhalation) (USP, Piramal Critical Care, Telangana, India) and decapitated. Brains were rapidly removed, chilled in ice‐cold phosphate‐buffered saline (PBS; Sigma‐Aldrich, St. Louis, MO, USA), and positioned in a mouse brain matrix (Adult 30 g, Coronal Sections; ASI Instruments, USA). A coronal section containing the SNc was isolated, and bilateral SNc tissue punches (0.50 mm; Precision Brain Punch, Ted Pella Inc., USA) were obtained and stored at −80°C. SNc tissue was homogenized in 100 μL of lysis buffer, which consisted of 1× RIPA buffer (Millipore Sigma, USA) and a protease inhibitor cocktail powder with EDTA (Sigma‐Aldrich, USA). The sample was centrifuged at 14000 RPM for 20 min at 4°C, and the supernatant collected. Protein concentrations were determined via Pierce BCA Protein Assay Kit (Thermo Fisher Scientific, Waltham, MA, USA). Samples were adjusted to a concentration of 30 μg of protein in 17 μL of supernatant using 2× Laemmli Sample Buffer (Bio‐Rad, USA) containing β‐mercaptoethanol (Bio‐Rad, USA), and denatured for 5 min at 95°C. Expression levels of FIS1 and DRP1 were determined using western blot analysis; 30 μg protein/lane and a Precision Plus Protein All Blue Prestained Protein Standard (Bio‐Rad, USA) were loaded onto a 16.5% Mini‐PROTEAN Tris/Tricine Precast Protein Gel (Bio‐Rad, USA) for detection of FIS1 and a 12% Mini‐PROTEAN TGX Precast Protein Gel (Bio‐Rad, USA) for DRP1. Gel electrophoresis was performed for 1 h at 100 V, followed by transfer for 1.5 h at 100 V at 4°C to 0.45 μm nitrocellulose membranes (Bio‐Rad, USA). Membranes were incubated for 1 h at room temperature with 5% nonfat dried milk dissolved in TBS‐T (10 mM Tris, 150 mM NaCl, and 0.5% Tween‐20) and then washed three times with TBS‐T (5 min per wash). The membranes were incubated overnight at 4°C with primary antibodies against FIS1 (Rabbit polyclonal antibody, 1:5000, GeneTex, USA) and β‐Actin (Mouse monoclonal antibody, 1:20000, Proteintech, USA) or DRP1 (Rabbit monoclonal antibody, 1:1000, Abcam, USA) and β‐Actin (Mouse monoclonal antibody, 1:20000, Proteintech, USA), with β‐Actin serving as the loading control. Incubation with primary antibodies was followed the next day by three washes with TBS‐T for 5 min each and incubation with fluorescent secondary antibodies (Goat anti‐rabbit Alexa Fluor 800 Plus, 1:20000, Invitrogen, USA, or Goat anti‐mouse Alexa Fluor 680, 1:10000, Invitrogen, USA) for 1 h at room temperature. Membranes were washed again three times with TBS‐T for 5 min each, after which they were imaged using a LI‐COR Odyssey IR Imager (LI‐COR Bio, Lincoln, NE, USA). Relative optical densities of FIS1 (17 kDa), DRP1 (83 kDa), and β‐actin (42 kDa) were determined using Image Studio Lite software.

### 2.3 Immunofluorescence and Mitochondrial Density

Mice (*n* = 4 per group) underwent chronic administration of saline, meth, rasagiline + saline, or rasagiline + meth after which subjects were administered ketamine (50 mg/kg; Dechra, Overland Park, KS, USA)/xylazine (4.5 mg/kg; Dechra, Overland Park, KS, USA) and transcardially perfused with 4% paraformaldehyde (PFA; Electron Microscopy Sciences, Hatfield, PA, USA) in PBS. Brains were extracted, post‐fixed overnight in 4% PFA in PBS, and cryoprotected in 30% sucrose in PBS. Fixed brains were sectioned at a thickness of 40 μm using a microtome (Leica SM2010R, Deerfield, IL, USA), and sections entailing SNc were collected. Every sixth section was stained for tyrosine hydroxylase (TH^+^; mouse monoclonal primary antibody, 1:200, Invitrogen, USA; donkey anti‐mouse IgG Alexa Fluor 488 secondary antibody, 1:200, Invitrogen, USA) to identify dopamine neurons, resulting in a total of six SNc sections per brain stained. Slices were counterstained for voltage‐dependent anion‐selective channel protein 1 (VDAC1^+^; rabbit polyclonal primary antibody, 1:200, Abcam, USA; donkey anti‐rabbit IgG Alexa Fluor 555 secondary antibody, 1:200, Invitrogen, USA), an outer mitochondrial membrane protein, to label mitochondria. Before incubating with primary antibodies, sections were treated with 20% formic acid (MilliporeSigma, Burlington, MA, USA) for antigen retrieval, then blocked with 5% normal donkey serum (MilliporeSigma, Burlington, MA, USA), 3% Triton X‐100 (Sigma‐Aldrich, St. Louis, MO, USA) in PBS + TWEEN‐20 (PBS‐T; Sigma‐Aldrich, St. Louis, MO, USA), and FAB fragment (Donkey Anti‐mouse IgG; 1:200, JacksonImmunoResearch, USA). Sections were washed with PBS‐T between steps. Stained sections were mounted on glass slides (Electron Microscopy Sciences, Hatfield, PA, USA) with ProLong Diamond Antifade Mountant (Invitrogen, USA), cover‐slipped, and stored at −20°C. High‐resolution *z*‐stacks (0.101 μm × 0.101 μm; 0.3 μm steps) of TH^+^ and VDAC1^+^ neurons in the SNc were acquired with multichannel image acquisition using an Apotome fluorescence microscope with a high‐magnification Plan‐Apochromat 63×/1.40, Oil DIC, ∞/0.17 objective (Zeiss, USA). Mitochondrial density measurements were conducted using Imaris MeasurementPro software with methods adapted from (Guzman et al. 2018). 3D reconstructions of the soma were generated using machine‐learning segmentation. The processes and nucleus of the neuron were removed using the “background labeling” option, and somatic boundaries defined using the “foreground labeling” option. The cytosolic volume was determined based on TH^+^ staining using the ‘detailed statistics’ feature for the somatic surface, and the volume occupied by mitochondria was determined based on VDAC1^+^ staining using the overlapping volume‐to‐surface ratio to determine relative mitochondrial mass per unit cytosol. Mitochondrial densities of 17–20 SNc dopamine neurons were quantified per mouse.

### 2.4 Quantitative Polymerase Chain Reaction (qPCR) and Mitochondrial DNA Copy Number

Chronic saline and meth‐treated mice (*n* = 10 per group) were decapitated under isoflurane anesthesia 1 day after the last saline/meth injection. Brains were rapidly removed, chilled in ice‐cold PBS, and a coronal section entailing the SNc isolated using a mouse brain matrix (Rodent Brain Matrix‐ Mouse, Adult 30 g, Coronal Sections; ASI Instruments, USA). A biopsy punch needle (Precision Brain Punch, 0.50 mm; Ted Pella Inc., USA) was used to collect bilateral tissue punches of the SNc, and the tissues were stored at −80°C. DNA was extracted from the frozen tissue using a Qiagen DNeasy Blood & Tissue Kit (Qiagen, Germantown, MD, USA) following standard protocol preparations, except that DNA was eluted in 50 μL of Buffer AE (10 mM Tris‐Cl, 0.5 mM EDTA; pH 9.0) following a 5‐min incubation at room temperature in the final elution step. DNA concentrations of the samples were measured using a NanoDrop 2000 spectrophotometer (Thermo Scientific, USA); 60 ng of DNA was used per qPCR. For each primer set (NADH dehydrogenase subunit 1 (ND1) and Hexokinase 2 (HK2)), a PCR master mix was prepared containing 1X SYBR Green (Applied Biosystems, USA) and 10 μM forward and reverse primers (Integrated DNA Technologies, USA). qPCR primers were designed based on previously published work (Quiros et al. 2017) (Table S1). The master mix was added to each DNA sample to reach a final volume of 15 μL. Each sample was run in triplicate. The qPCR cycle threshold (Ct) values for each sample were determined using the QuantStudio 3 Real Time PCR System (Applied Biosystems, USA). The ΔCt was calculated between nuclear and mitochondrial DNA genes as follows: ΔCt = Ct (nuclear DNA gene) – Ct (mitochondrial DNA gene), and mtDNA copy number was calculated for both saline and meth‐treated groups using the following formula: mtDNA copy number = 2 × 2^ΔCt^ and normalized to the average saline group.

### 2.5 High‐Resolution Respirometry and Fluorometry

Oxygen Consumption Rates (OCRs) and hydrogen peroxide (H_2_O_2_) were simultaneously measured in ex vivo SNc tissue from mice administered either saline or meth (5 mg/kg) for 28 consecutive days using the Oroboros NextGen‐O2k Fluo system (Oroboros Instruments, Innsbruck, Austria). Methods for examining OCRs and H_2_O_2_ were consistent with Logan et al. (2024), with OCRs measured using high‐resolution respirometry and H_2_O_2_ measured simultaneously by fluorometry. Daily calibration of the H_2_O_2_ signal was performed by sequential injections of 0.1 μM H₂O₂ to generate a standard curve (0–0.5 μM). Measurements were conducted in assay buffer consisting of buffer Z (containing 105 mM K‐MES, 30 mM KCl, 10 mM K_2_HPO_4_, 5 mM MgCl_2_.6H_2_O, 0.5 mg/mL bovine serum albumin at pH 7.1) with 1 mM EGTA, 20 mM creatine monohydrate, 10 μM Amplex UltraRed (AmR), 1 U/mL horseradish peroxidase (HRP), and 5 U/mL superoxide dismutase (SOD) at 37°C. After acquiring the H_2_O_2_ standard curve, air calibration was performed using the DatLab protocol “O_2_‐calibration air.” One day after the last saline/meth injection, mice (*n* = 10 mice per group) were anesthetized using isoflurane (up to 5% inhalation), decapitated, and brains rapidly removed. Extracted brains were placed in a mouse brain matrix, and a coronal section entailing the SNc was isolated. Bilateral samples of SNc tissue were collected, and wet tissue weight recorded; average total SNc wet tissue weight was 3.6 ± 0.5 mg per mouse. Tissue was suspended in ice‐cold Buffer X (7.23 mM K_2_EGTA, 2.77 mM CaK_2_EGTA, 20 mM imidazole, 0.5 mM DTT, 20 mM taurine, 5.7 mM ATP, 14.3 mM phosphocreatine disodium salt hydrate, 6.56 mM MgCl_2_.6H_2_O, and 50 mM K‐MES at pH 7.1) and permeabilized with saponin (50 μg/mL) for 30 min, followed by three 5 min washes in ice‐cold buffer Z at 4°C. Bilateral tissue punches from a single mouse were permeabilized together and loaded into the same Oroboros chamber for experimentation; individual datapoints reflect outcomes from the total SNc tissue collected from a single mouse, not an average of two experiments. OCRs and H_2_O_2_ levels were determined by sequential addition of substrates/inhibitors using Hamilton microsyringes as follows: glutamate (G, 10 mM) + malate (M, 2 mM) (G/M), pyruvate (P, 5 mM) + malate (M, 2 mM) (P/M), ADP (2.5 mM), succinate (Suc, 10 mM), cytochrome C (CyC, 4 mM), rotenone (Rot, 0.5 μM), antimycin A (AA, 5 μM) and ascorbate (A, 2 mM) + N, N, N′, N′‐tetramethyl‐p‐phenylenediamine (T, 0.5 mM) (A/T). Glutamate, malate and pyruvate are complex I substrates, ADP is a precursor to oxidative phosphorylation (OXPHOS), succinate is a complex II substrate, cytochrome C is an electron carrier for complex III to IV, rotenone is a complex I inhibitor, antimycin A is a complex III inhibitor and ascorbate and N, N, N′, N′‐tetramethyl‐p‐phenylenediamine (TMPD) are complex IV substrates. OXPHOS capacity refers to the mitochondria's maximum ability to produce ATP. Complex I and complex I + II OXPHOS capacities were calculated by subtracting leak state respiration (C_L_, derived from glutamate and malate addition) from complex‐linked active phosphorylation respiration (C_P_; determined after addition of ADP and succinate) (Rose et al. 2024). Coupling efficiency indicates how effectively mitochondria use oxygen, that is, how well electrons are transferred through the electron transport chain, rather than being lost as leak or uncoupled respiration. Complex I and complex I + II coupling efficiencies were calculated using the formula 1‐C_P_/C_L_ (Logan et al. 2023). OCRs and H_2_O_2_ measurements were first calculated based on milligrams of wet brain tissue using DatLab software (Oroboros Instruments, Innsbruck, Austria). Mitochondrial oxidative stress is necessary for chronic meth‐induced degeneration, as evident by the mitochondrial antioxidant mitoTEMPO being neuroprotective (Du et al. 2021). Citrate synthase activity is often used as an index of mitochondrial content; however, the activity of this enzyme can be decreased by oxidative modification (Chepelev et al. 2009). Therefore, measurements acquired using tissue from mice treated with chronic meth were normalized to account for chronic meth‐induced degeneration of TH^+^ SNc neurons (Du et al. 2021; Pilski and Graves 2023) and the observed increase in mitochondrial density in TH^+^ SNc neurons (Section 3.2), rather than to citrate synthase activity. Our prior studies examining chronic meth‐induced SNc degeneration result in a mean decrease of TH^+^ SNc neurons by 23.80% (Du et al. 2021; Pilski and Graves 2023), corresponding to a neuronal scaling factor of 0.7620 (meth/saline); chronic meth administration also increased mitochondrial density in TH^+^ SNc neurons by 64.55% (current study, Section 3.2), yielding a mitochondrial density scaling factor of 1.6455 (meth/saline). The composite normalization for tissue from meth‐treated mice was derived as:

$$\begin{array}{c} \begin{array}{c} {\mathrm{OCR}}_{\text{normalized meth}}={\mathrm{OCR}}_{\text{raw meth}}÷\left(\text{neuronal factor}\times \text{mitochondrial density factor}\right)\\ ={\mathrm{OCR}}_{\text{raw meth}}÷\left(0.7620\times 1.6455\right)\\ ={\mathrm{OCR}}_{\text{raw meth}}÷1.2538 \end{array} \end{array}$$

### 2.6 Statistical Analysis

Statistical analyses were performed using Prism version 11.0.1 (GraphPad Software, La Jolla, CA, USA) or MATLAB version R2026a (MathWorks, Natick, MA, USA). No pre‐estimation of sample sizes was performed; sample sizes were guided by pilot studies. The ROUT method (*Q* = 1%) was used for outlier detection, with 2 outliers identified and removed from experiments quantifying mitochondrial DNA copy number (2 data points from chronic meth‐treated mice). For mitochondrial fission, mitochondrial DNA copy number, respirometry, and fluorometry experiments, datasets were tested for normality using the Shapiro–Wilk test and analyzed using unpaired Student's *t*‐ or Mann–Whitney tests as appropriate (Table S2). For mitochondrial density experiments, quantification was performed by an experimenter blinded to treatment history and data first analyzed using a nested one‐way ANOVA with Tukey's *post‐hoc* or nested *t*‐test followed by a secondary analysis wherein total neurons analyzed per experimental subject were averaged and data analyzed using ordinary one‐way ANOVA with Tukey's *post hoc* or unpaired *t*‐test. Data are presented as scatter plots and/or histograms, or as nested scatter plots, with the mean and standard error of the mean depicted; *α* = 0.05. Detailed statistical reporting is presented in the figure legends for all datasets and Table S3 provides a list of reagents, antibodies, and instruments used in the current study.

## 3 Results

### 3.1 Chronic Methamphetamine Administration Decreased Expression of Mitochondrial Fission 1 (FIS1) and Dynamin‐Related Protein 1 (DRP1) in the Substantia Nigra Pars Compacta

**FIGURE 1:**
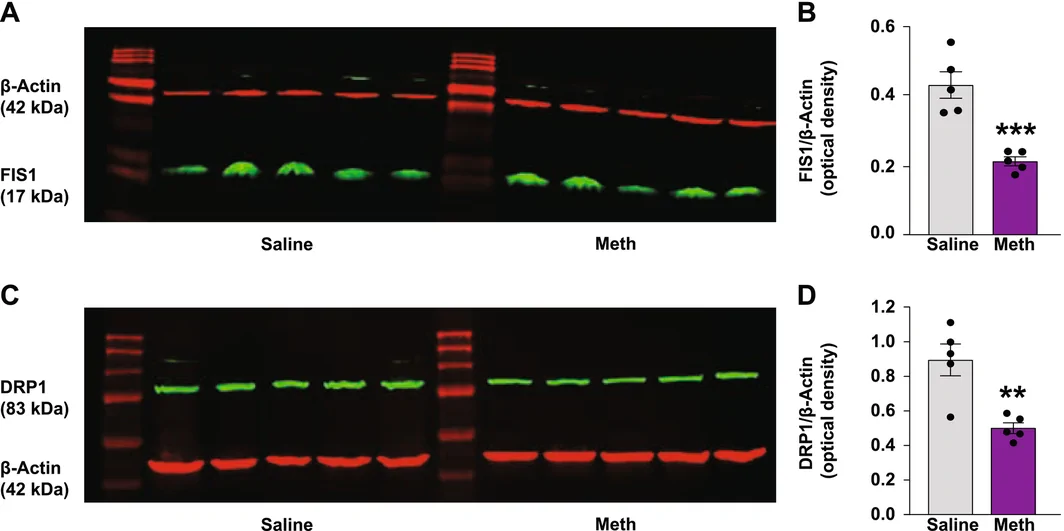
Chronic meth administration decreased FIS1 and DRP1 expression in the SNc. Male mice were administered saline or meth for 28 days, after which FIS1 and DRP1 expression in the SNc was quantified. (A) Western blot illustrating SNc expression of FIS1 (17 kDa; green) in male mice treated with chronic saline or meth. β‐Actin (42 kDa; red) was used as a loading control. (B) SNc expression of FIS1 was decreased in meth‐treated mice (*n* = 5) compared to saline‐treated mice (*n* = 5). Data analyzed using unpaired *t*‐test (*t*(8) = 5.468, ***p = 0.0006, two‐tailed). (C) Western blot comparing SNc expression of DRP1 (83 kDa; green) in male mice treated with saline or meth. β‐Actin (42 kDa; red) was used as a loading control. (D) SNc expression of DRP1 was decreased in meth‐treated mice (*n* = 5) compared to saline‐treated mice (*n* = 5). Data analyzed by unpaired *t*‐test (*t*(8) = 4.102, **p = 0.0034, two‐tailed); histograms illustrate the mean ± standard error of the mean.

To determine whether chronic in vivo meth administration alters expression of mitophagy‐associated FIS1 and/or DRP1 proteins in the SNc, mice were administered saline (10 mL/kg; i.p.) or meth (5 mg/kg; i.p.) for 28 days, and FIS1 and DRP1 protein expression in the SNc examined. Densitometric quantification indicated that expression of both FIS1 (Figure 1A,B) and DRP1 (Figure 1C,D) in the SNc was decreased by chronic meth administration, with FIS1 expression decreased by approximately 2‐fold and DRP1 expression decreased by approximately 1.8‐fold.

### 3.2 Chronic Methamphetamine Administration Resulted in a Monoamine Oxidase‐Dependent Increase in Mitochondrial Density in Substantia Nigra Pars Compacta Dopamine Neurons

**FIGURE 2:**
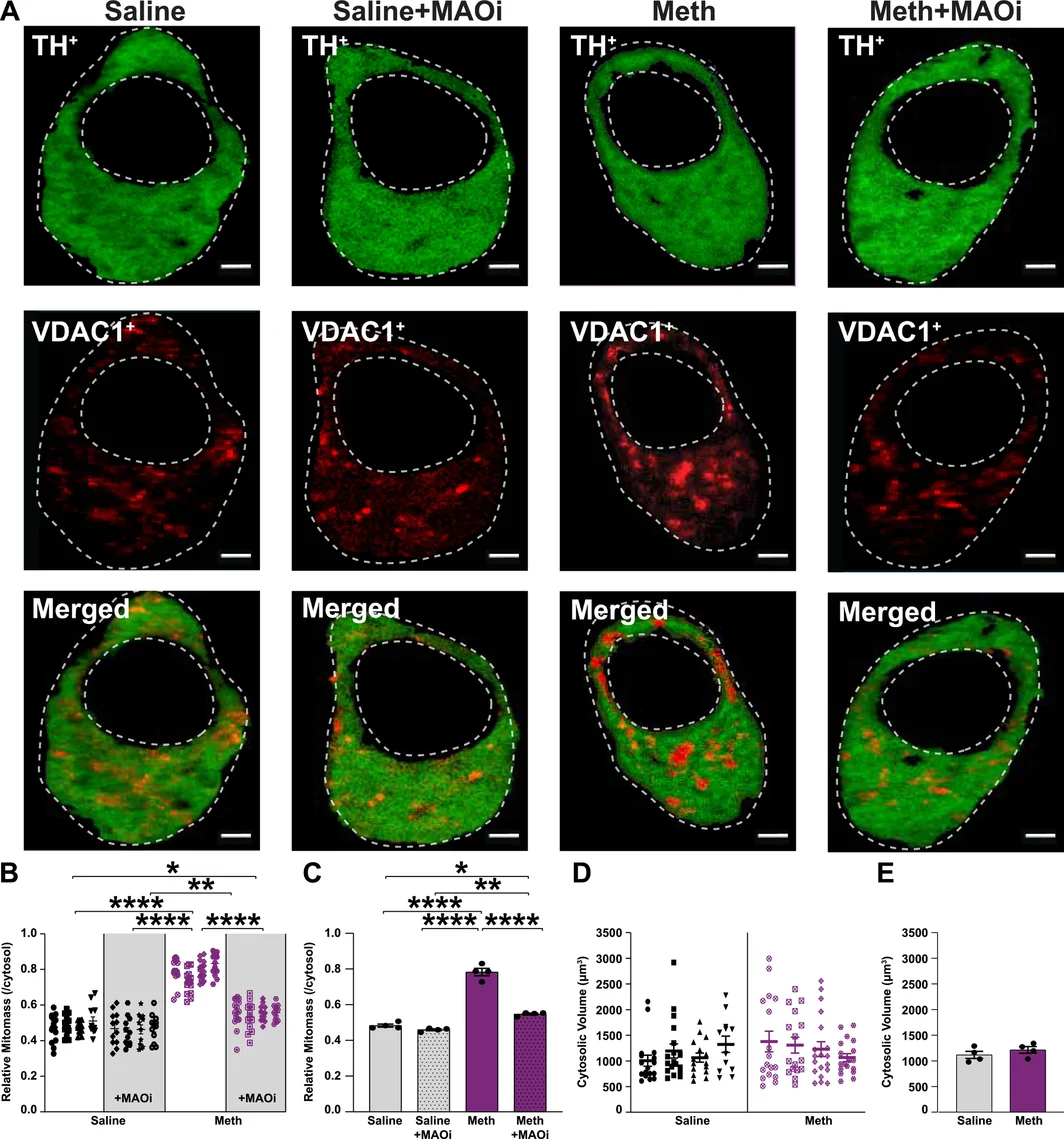
Chronic meth administration resulted in an MAO‐dependent increase in SNc dopamine neuron mitochondrial density. Male mice were administered saline, meth, saline + rasagiline (MAOi), or meth + MAOi for 28 days, after which mitochondrial density in SNc dopamine neurons was quantified. (A) Representative high‐magnification images of SNc dopamine neurons showing tyrosine hydroxylase staining (TH^+^; green; top), mitochondrial labeling with voltage‐dependent anion channel 1 (VDAC1^+^; red; middle), and merged images (bottom) with a white dashed line marking the cell and nuclear membranes. Images were processed using Imaris MeasurementPro software, and the presented images are a single 2D plane from a 3D‐reconstructed soma of a mouse treated with saline, meth, and meth + MAOi; scale bars denote 3 μm. (B) Chronic meth administration increased the mitochondrial density in SNc dopamine neurons and this increase was prevented by pretreatment with the MAO inhibitor rasagiline (+MAOi) whereas +MAOi pretreatment had no effect in saline‐treated mice; saline: *n* = 62 neurons/4 mice, saline + MAOi: *n* = 48 neurons/4 mice, meth: *n* = 70 neurons/4 mice, and meth + MAOi: *n* = 52 neurons/4 mice. Data analyzed using nested one‐way ANOVA (*F*_(3,12)_ = 147.8, ****p < 0.0001) with Tukey's *post hoc* analysis (saline vs. meth, ****p < 0.0001; saline + MAOi vs. meth + MAOi, **p = 0.0022; saline vs. saline + MAOi, *p* = 0.5993; saline vs. meth + MAOi, *p = 0.0152; meth vs. saline + MAOi, ****p < 0.0001; meth vs. meth + MAOi, ****p < 0.0001). Error bars depict the mean ± standard error of the mean. (C) A secondary analysis was performed wherein neurons per mouse were averaged; results from the secondary analysis recapitulate findings using nested analyses. Data analyzed using ordinary one‐way ANOVA (*F*_(3,12)_ = 154.6, ****p < 0.0001) with Tukey's *post hoc* analysis (saline vs. meth, ****p < 0.0001; saline + MAOi vs. meth + MAOi, **p = 0.0011; saline vs. saline + MAOi, *p* = 0.5193; saline vs. meth + MAOi, *p = 0.0114; meth vs. saline + MAOi, ****p < 0.0001; meth vs. meth + MAOi, ****p < 0.0001). (D) No differences were observed in the cytosolic volume of SNc dopamine neurons between the saline (*n* = 62 neurons/4 mice) and meth‐treated group (*n* = 70 neurons/4 mice). Data analyzed using nested *t*‐test (Saline: *M* = 1136, SD = 491.9, Meth: *M* = 1242, SD = 620.1, *t*(6) = 1.079, *p* = 0.3222 with Cohen's *d* effect size of 0.7037); error bars indicate the mean ± standard error of the mean. (E) A secondary analysis was performed wherein neurons per mouse were averaged; results from the secondary analysis recapitulate findings using nested analyses. Data analyzed using unpaired *t*‐test (*t*(6) = 1.011, *p* = 0.3510, two‐tailed). Histograms illustrate the mean ± standard error of the mean.

A deficit in mitophagy, as suggested by decreased expression of proteins involved in mitophagy (Figure 1), would presumably result in an accumulation of mitochondria. To test whether this was the case in SNc dopamine neurons, mitochondrial density was quantified. Chronic in vivo meth administration increased mitochondrial density in SNc dopamine neurons by approximately 60% (Figure 2B). Moreover, this increase in mitochondrial density was MAO‐dependent. Pretreating mice with the MAO‐B inhibitor rasagiline (+MAOi) had no impact on mitochondrial density in saline‐treated mice but attenuated the change in density in meth‐treated mice (Figure 2B). However, the attenuation was incomplete, with density being slightly higher in SNc dopamine neurons from meth + MAOi relative to saline and saline + MAOi‐treated mice. A secondary analysis was performed wherein neurons were averaged per mouse; this secondary analysis similarly shows chronic in vivo meth administration resulted in a monoamine oxidase‐dependent increase in mitochondrial density (Figure 2C). Importantly, chronic meth administration had no impact on the cytosolic volume of SNc dopamine neurons (Figure 2D,E). This suggests that the change in mitochondrial density is due to mitochondrial changes rather than simply a shrinkage of neurons.

To complement mitochondrial density studies, mitochondrial DNA (mtDNA) copy number in the SNc was also assessed by qPCR. Chronic meth administration also increased SNc mitochondrial DNA copy number (Figure S2). Altogether, decreased expression of mitophagy‐associated proteins (Figure 1), increased mitochondrial density (Figure 2), and increased mtDNA copy number (Figure S2) provide converging lines of evidence suggesting that chronic meth administration impairs mitophagy, resulting in an accumulation of mitochondria in the SNc.

### 3.3 Chronic Methamphetamine Administration Impaired Mitochondrial Function in the Substantia Nigra Pars Compacta

**FIGURE 3:**
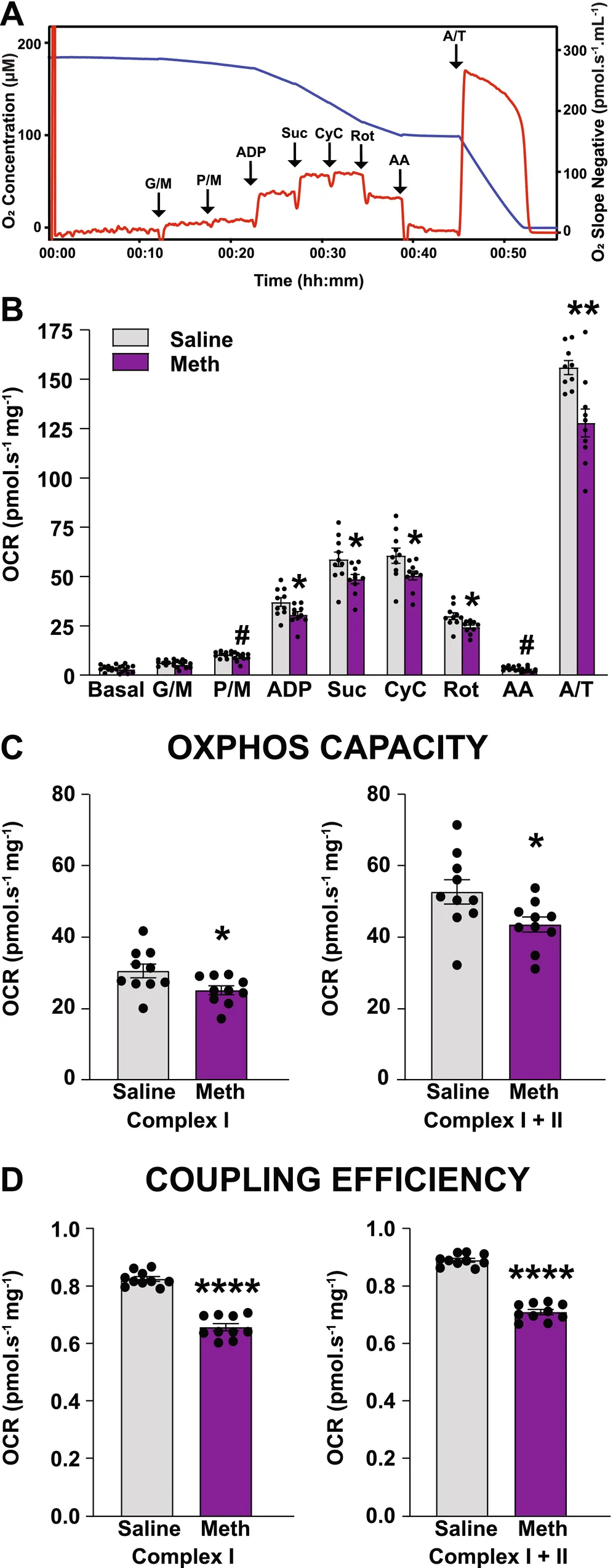
Chronic meth administration decreased mitochondrial oxygen consumption rates in the SNc. High‐resolution respirometry was used to measure mitochondrial respiration in ex vivo SNc tissue. (A) Sample trace depicting an experiment measuring oxygen consumption rates (OCRs) after adding different substrates and inhibitors. The red line indicates the OCR, represented as the O_2_ slope negative, measured in pmol.s^−1^.mL^−1^, graphed on the right *y*‐axis, and the blue line indicates the O_2_ concentration, measured in μM, graphed on the left *y*‐axis. Abbreviations used in representative traces are: Glutamate + Malate (G/M), Pyruvate + Malate (P/M), Adenosine diphosphate (ADP), Succinate (Suc), Cytochrome C (CyC), Rotenone (Rot), Antimycin A (AA), Ascorbate + *N*, N, N′, N′‐tetramethyl‐p‐phenylenediamine (A/T). Oxygen consumption rates (OCRs) were assessed in freshly dissected SNc tissue from mice chronically administered either saline (*n* = 10) or meth (*n* = 10). (B) Mitochondrial OCRs were unchanged under basal conditions (*t*(18) = 0.7044, *p* = 0.4902, two‐tailed) and after addition of G/M (*t*(18) = 1.306, *p* = 0.2081, two‐tailed). There was a trend of decreased OCR in SNc tissue from chronic meth‐treated mice after addition of P/M (*t*(18) = 1.771, ^#^ *p* = 0.0935, two‐tailed) and a decrease after adding ADP (*t*(18) = 2.367, *p = 0.0293, two‐tailed), Suc (*t*(18) = 2.350, *p = 0.0304, two‐tailed), CyC (*t*(18) = 2.257, *p = 0.0367, two‐tailed), and Rot (*t*(18) = 2.763, *p = 0.0128, two‐tailed), a trend after AA (*t*(18) = 1.930, ^#^ *p* = 0.0695, two‐tailed) addition and a decrease with A/T (*t*(18) = 3.429, **p = 0.0032) addition. (C) Complex I (left) and complex I + II (right) OXPHOS capacities were decreased in SNc tissue from mice treated with meth for 28 days (*n* = 10) compared to SNc tissue from mice treated with saline (*n* = 10). Data analyzed using unpaired *t*‐test (Complex I OXPHOS capacity: *T*(18) = 2.378, *p = 0.0287, two‐tailed; Complex I + II OXPHOS capacity: *T*(18) = 2.288, *p = 0.0345). (D) Complex I (left) and complex I + II (right) coupling efficiencies were decreased in SNc tissue from mice treated with meth for 28 days (*n* = 10) compared to SNc tissue from saline‐treated mice (*n* = 10). Data analyzed using unpaired *t*‐test (Complex I coupling efficiency: *t*(18) = 11.31, ****p < 0.0001, two‐tailed; Complex I + II coupling efficiency: *t*(18) = 16.02, ****p < 0.0001). Histograms illustrate the mean ± standard error of the mean.

Mitochondrial function was examined using high‐resolution respirometry in ex vivo SNc tissue collected from mice treated with either chronic saline or meth; experimentation was performed 1 day after the last saline/meth injection. A representative trace illustrating OCR measurements is provided in Figure 3A. OCRs in SNc tissue from mice treated with chronic meth were normalized to account for chronic meth‐induced SNc degeneration and increased mitochondrial density in SNc dopamine neurons. Chronic meth administration did not alter basal OCRs, nor were there differences observed following the addition of complex I substrates (glutamate + malate). However, OCRs were decreased in SNc tissue from chronic meth‐treated mice after the addition of ADP, the complex II substrate (succinate), the electron carrier (cytochrome c), the complex I inhibitor (rotenone), and the complex IV substrates (ascorbate + *N*, N, N′, N′‐tetramethyl‐p‐phenylenediamine) (Figure 3B). Trends were also observed after complex I substrates pyruvate + malate as well as the complex III inhibitor antimycin A were added (Figure 3B). Complex I and complex I + II OXPHOS capacities (Figure 3C) as well as efficiencies (Figure 3D) were all decreased by approximately 20% in SNc tissue from chronic meth‐treated mice. Altogether, the presented results suggest that chronic in vivo meth administration impairs SNc mitochondrial function.

### 3.4 Chronic Methamphetamine Administration Had no Effect on H_2_O_2_ Levels in the Substantia Nigra Pars Compacta

**FIGURE 4:**
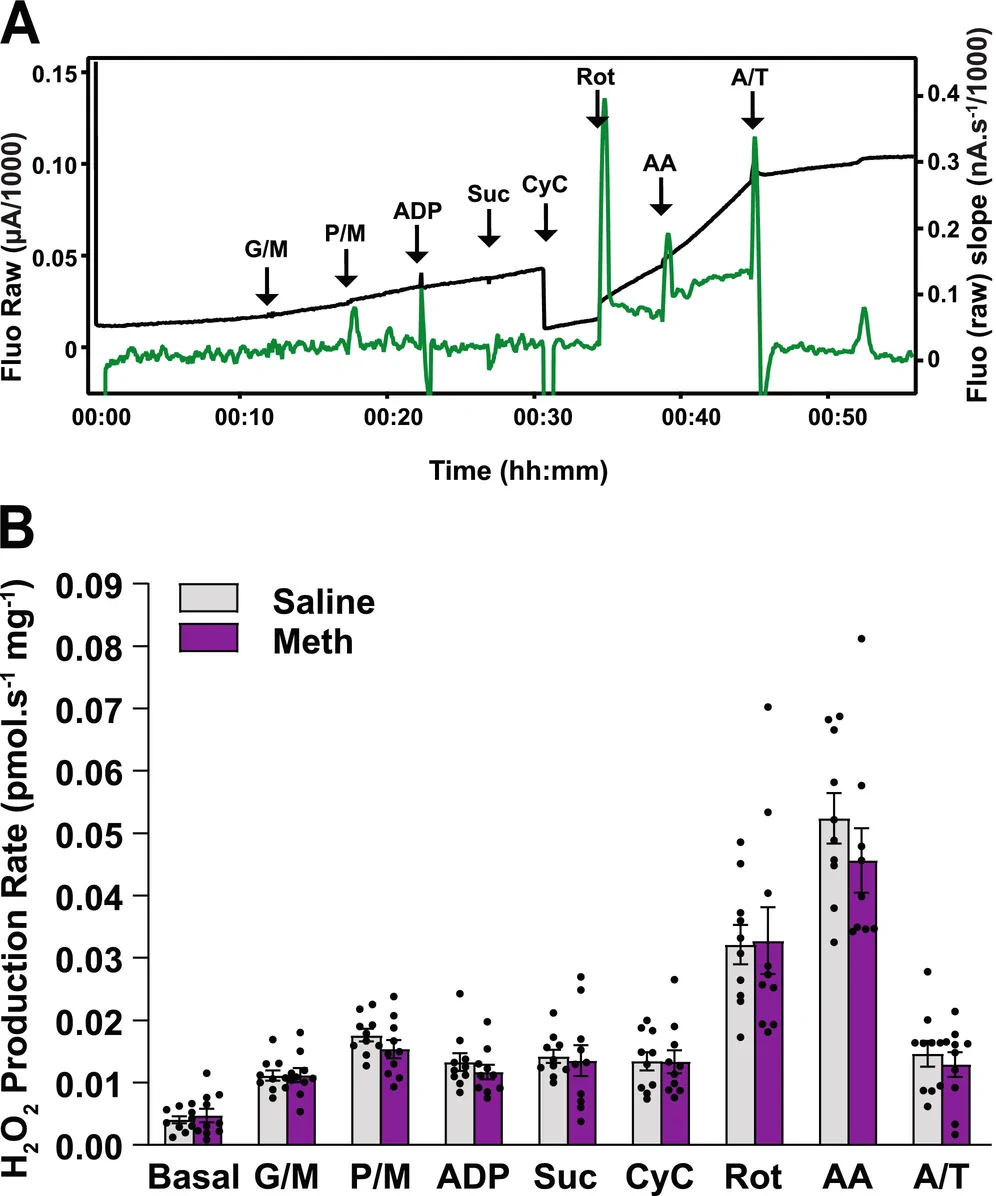
Chronic meth administration had no effect on mitochondrial H_2_O_2_ levels in the SNc. (A) Sample trace showing measurement of H_2_O_2_ levels following the addition of substrates and inhibitors. The green line indicates the H_2_O_2_, represented as the Fluo (raw) slope measured in nA.s^−1^/1000, graphed on the right y‐axis, and the black line indicates the H_2_O_2_ cumulative concentration, measured in μA/1000, graphed on the left y‐axis. Abbreviations used in representative traces are: Glutamate + Malate (G/M), Pyruvate + Malate (P/M), Adenosine diphosphate (ADP), Succinate (Suc), Cytochrome C (CyC), Rotenone (Rot), Antimycin A (AA), Ascorbate + *N*, N, N′, N′‐tetramethyl‐p‐phenylenediamine (A/T). (B) Fluorometry and oxygen consumption rates (Figure 3) were performed simultaneously in the same tissue. Chronic meth administration (*n* = 10) had no impact on ex vivo SNc basal H_2_O_2_ compared to saline‐treated mice (*n* = 10) (*t*(18) = 0.5558, *p* = 0.5852, two‐tailed) or after addition of G/M (*t*(18) = 0.06334, *p* = 0.9502, two‐tailed), P/M (*t*(18) = 1.254, *p* = 0.2258, two‐tailed), ADP (*U* = 93, *p* = 0.3930, two‐tailed), Suc (*t*(18) = 0.2475, *p* = 0.8073, two‐tailed), CyC (*t*(18) = 0.01967, *p* = 0.9845, two‐tailed), Rot (*U* = 100, *p* = 0.7394, two‐tailed), AA (*U* = 74, *p* = 0.2110, two‐tailed) or A/T (*t*(18) = 0.5969, *p* = 0.5580, two‐tailed). Data analyzed using unpaired *t*‐test for datasets that passed normality testing and Mann–Whitney test for datasets failing normality testing; histograms illustrate the mean ± standard error of the mean.

H_2_O_2_ levels in ex vivo SNc tissue were measured using fluorometry with tissue collected 1 day after chronic saline or meth treatment (meth was not present in tissue at the time of experimentation). A representative trace illustrating H_2_O_2_ measurements is provided in Figure 4A. There was no difference in H_2_O_2_ under basal conditions or after the addition of substrates/inhibitors in SNc tissue from chronic saline or meth‐treated mice (Figure 4B).

## 4 Discussion

The goal of the present study was to examine the impact of chronic in vivo meth administration on mitochondria in the SNc. Experimental outcomes provide evidence that chronic meth administration, which results in SNc degeneration (Du et al. 2021; Graves et al. 2021; Pilski and Graves 2023), decreases SNc expression of fission and mitophagy‐associated proteins FIS1 and DRP1. This suggests potential deficits in the ability to clear damaged mitochondria. Consistent with this, our results also demonstrate that chronic meth administration increased mitochondrial density in SNc dopamine neurons and that this increase was MAO‐dependent. Prior studies demonstrate that chronic meth‐induced SNc degeneration is also MAO‐dependent (Du et al. 2021; Graves et al. 2021; Pilski and Graves 2023), suggesting a link between meth‐induced SNc degeneration and increased mitochondrial density. To determine whether chronic meth administration adversely impacted mitochondrial function, high‐resolution respirometry was performed in SNc tissue collected from mice treated with saline or meth. Mitochondrial OCRs obtained by most of the complex substrates/inhibitors, complex I and I + II OXPHOS capacities, as well as coupling efficiencies, were decreased in SNc tissue from mice treated with chronic meth. Altogether, presented results suggest that chronic in vivo meth administration results in an accumulation of functionally compromised mitochondria in the SNc, which may contribute to a bioenergetic insufficiency and chronic meth‐induced neurodegeneration.

### 4.1 Methamphetamine, Mitophagy, and Mitochondrial Density

Mitochondrial quality control is maintained through coordinated cycles of fission, fusion, and mitophagy, allowing damaged mitochondrial segments to be segregated and selectively degraded (Ashrafi and Schwarz 2013). Fission is an early step in mitophagy, which generates smaller mitochondrial fragments that are more readily recognized and engulfed by autophagic machinery. DRP1 contributes to this process and is recruited from the cytosol to the mitochondrial outer membrane by adaptor proteins such as FIS1 (Losón et al. 2013). Upon recruitment, DRP1 oligomerizes and constricts the membrane (Francy et al. 2015; Mears et al. 2011), facilitating the segregation of damaged mitochondrial components for selective degradation (Egner et al. 2022; Nolden et al. 2023). Changes to this fission machinery have been shown to impair mitochondrial turnover (Yue et al. 2015) and promote the persistence of dysfunctional mitochondria (Benard and Rossignol 2008; Calkins et al. 2011; Feng et al. 2021; Shi et al. 2023).

In the present study, chronic in vivo meth administration decreased SNc expression of both DRP1 and FIS1. These findings are consistent with in vitro work by Lenzi et al. (2022), who show that meth decreases cytosolic and mitochondrial levels of DRP1 and FIS1 in PC12 cells. Deficits in proteins that are involved in helping to drive the clearance of damaged mitochondria would presumably result in an accumulation of mitochondria and/or perhaps an increase in mitochondrial size. The current study demonstrates an increase in mitochondrial density in SNc dopamine neurons and Lenzi et al. (2022) show disrupted cristae and increased mitochondrial size in PC12 cells. Together, these pieces of evidence support the framework of meth resulting in an accumulation of mitochondria, likely due to impaired mitophagy. Importantly, in the present study, cytosolic volume was unchanged by chronic meth administration, indicating that the observed increase in density reflects altered mitochondrial abundance and/or size rather than cellular atrophy. The mechanisms underlying decreased DRP1 and FIS1 expression are unclear. However, we speculate that mitochondrial oxidative stress underlies this change in protein expression. While we did not observe a change in H_2_O_2_ in the SNc in the current report, this measurement was taken from ex vivo tissue collected 1 day after the last saline/meth injection. Meth was therefore no longer present at the time of tissue collection. However, when meth is present, that is, during chronic meth administration, meth induces MAO‐dependent mitochondrial oxidative stress, which is necessary for degeneration of SNc dopamine neurons (Du et al. 2021; Graves et al. 2020; Pilski and Graves 2023). Similarly, MAO inhibition prevented the change in mitochondrial density, indicating that the accumulation of mitochondria was MAO‐dependent. Altogether, these lines of evidence suggest the observed protein dynamics are likely due to mitochondrial oxidative stress. Future studies aimed at further exploring the relationship between chronic meth use, oxidative stress, and the expression of mitophagy‐associated proteins would be beneficial, as would studies to determine whether changes in protein expression result from protein degradation and/or changes in synthesis.

While the results are consistent with mitochondrial accumulation due to impaired clearance, it is possible that increased mitochondrial biogenesis could also contribute. However, given that the increase in density was MAO‐dependent and meth induces MAO‐dependent mitochondrial oxidative stress (Du et al. 2021; Graves et al. 2021, 2020) and SNc degeneration (Du et al. 2021; Graves et al. 2021; Pilski and Graves 2023), this is unlikely. Moreover, results indicate that mitochondrial function is also impaired after chronic meth. This suggests that the increased mitochondrial density is more likely due to the accumulation of damaged mitochondria rather than increased biogenesis, which would result in new, presumably functional mitochondria. Consistent with this interpretation, Moszczynska and Yamamoto (2011) show that an acute meth binge (10 mg/kg i.p. meth administered at 2 h intervals for a total of 4 injections) oxidatively modifies Parkin, thereby impairing its E3 ubiquitin ligase, proteasomal, and mitophagic function, which resulted in an accumulation of ubiquitinated proteins. Although our study did not examine PINK1 or Parkin, the observed decrease in DRP1 and FIS1 expression provides an additional upstream mechanism by which mitophagy may be adversely impacted by meth.

### 4.2 Methamphetamine and Mitochondrial Function

SNc dopamine neurons are particularly vulnerable to degeneration, with loss of SNc dopamine neurons being a hallmark feature of Parkinson's disease (Damier et al. 1999; Giguère et al. 2018; Kamath et al. 2022; Surmeier et al. 2017). We have similarly reported that SNc dopamine neurons are vulnerable to chronic meth‐induced degeneration (Du et al. 2021). The vulnerability of SNc dopamine neurons to degeneration seems to stem, at least in part, from mitochondria and mitochondrial oxidative stress (Du et al. 2021; Graves et al. 2021; Ni and Ernst 2022; Pacelli et al. 2015; Ricke et al. 2020; Surmeier et al. 2017). Of particular note, within the context of the current study is the bioenergetic demand of SNc dopamine neurons. SNc dopamine neurons have a highly branched and extensive axonal arbor, resulting in a complex network of synaptic terminals. This extensive architecture is thought to contribute to a high bioenergetic demand needed to sustain function (Pacelli et al. 2015). Results in the current report indicate that chronic meth administration results in impaired mitochondrial function. This mitochondrial dysfunction may drive a bioenergetic deficit that could contribute to degeneration.

Meth induces MAO‐dependent mitochondrial oxidative stress, which appears to be a necessary driver of SNc degeneration (Du et al. 2021; Graves et al. 2021; Pilski and Graves 2023). In the current report, the increase in SNc dopamine neuron mitochondrial density was also MAO‐dependent and the increased density was associated with impaired SNc mitochondrial function. Potential mechanisms underlying the observed mitochondrial dysfunction are unclear. Existing literature indicates that meth alters the abundance of electron transport chain complex components (Bazylianska et al. 2021). In isolated striatal mitochondria from rats, meth decreases subunit expression from multiple complexes, including complex I NADH dehydrogenase ubiquinone iron–sulfur protein 3 (NDUFS3), complex II Succinate Dehydrogenase Complex Flavoprotein Subunit A (SDHA), and complex III Ubiquinol‐cytochrome c reductase core protein II (UQCRC2) (Bazylianska et al. 2021). An acute meth binge (10 mg/kg meth administered at 2 h interval for a total of 4 injections) in rodent models also decreases complex II (succinate dehydrogenase) and complex II‐III activity in striatal mitochondria (Brown et al. 2005). Collectively, these studies complement our current findings of decreased complex I and complex I + II OXPHOS capacities and coupling efficiencies and potentially point to mechanisms that may underlie the observed impairment in mitochondrial function.

### 4.3 Methamphetamine and Mitochondrial Oxidative Stress

In the present study, H_2_O_2_ was measured in ex vivo SNc tissue using fluorometry. Despite changes in mitochondrial function resultant from chronic in vivo meth administration, there were no changes in H_2_O_2_ detected. Similarly, Dominguez‐Lopez et al. (2023) found no change in H_2_O_2_ in ex vivo mesolimbic tissue from mice that self‐administered meth relative to tissue from naive mice. In contrast, mitochondrial oxidative stress is reported to be increased in SNc dopamine neurons after repeated meth administration (Graves et al. 2021). While these results are seemingly at odds, the disparity is likely a consequence of methodological differences. In the study reporting increased mitochondrial oxidative stress (Graves et al. 2021), experiments were conducted in live ex vivo brain slices using the redox biosensor roGFP targeted to the mitochondrial matrix of SNc dopamine neurons. In contrast, experiments in the current report and in Dominguez‐Lopez et al. (2023) were performed using fluorometry with permeabilized ex vivo tissue. This is a salient difference given that the mitochondrial oxidative stress measured in the somatic compartment in Graves et al. (2021) was sensitive to L‐type calcium channel inhibition. Given that L‐type calcium channels are voltage‐gated, the functionality of these channels and mitochondrial consequences would no longer be observable in permeabilized tissue, wherein the electrochemical gradient is disrupted.

## 5 Limitations of the Current Study

Experimental outcomes presented in the current study provide converging pieces of evidence that are consistent with a model in which chronic in vivo meth administration impairs mitophagy, resulting in an accumulation of dysfunctional mitochondria. However, it is important to note study limitations. First, while the combination of decreased SNc FIS1 and DRP1 expression, increased mitochondrial density, and increased mitochondrial DNA copy number are consistent with impaired mitophagy, the experiments performed do not provide direct evidence of this. Both FIS1 and DRP1 are proteins involved in fission, which is associated with, and typically precedes mitophagy, although fission can occur independently of mitophagy (Ashrafi and Schwarz 2013; Kleele et al. 2021; Twig et al. 2008). Additionally, changes in mitochondrial density were determined based on volumetric assessments of VDAC1 immunofluorescence. We have interpreted the change as a change in mitochondrial density; however, an alternative would be a potential change in the amount of VDAC1 expression with no change in the amount or size of mitochondria. Given that we also observed an increase in mitochondrial DNA copy number, experimental outcomes are more consistent with a change in mitochondrial density. Future studies directly focused on examining the impact of meth on mitophagy and mitochondrial dynamics would be beneficial. A second limitation is that the high‐resolution respirometry data were obtained using bulk tissue. This is an important limitation, as the collected tissue punches would also contain other cell types, such as astrocytes. Astrocytes also express MAO enzymes, and it is unknown whether meth administration similarly induces MAO‐dependent mitochondrial oxidative stress in astrocytes. Because increasing astrocytic MAO‐B expression results in SNc degeneration (Mallajosyula et al. 2008; Siddiqui et al. 2011), astrocytes are likely contributing to chronic meth‐induced degeneration. Consequently, future studies are needed to address this issue. It is also important to note that H_2_O_2_ measurements in the current study are not just a measure of H_2_O_2_ production but are also influenced by removal and clearance by antioxidant systems present. Future studies examining potential meth effects on detoxification systems and/or antioxidant pathways would therefore be of value. Lastly, our studies were focused on male subjects based on prior evidence showing that SNc dopamine neurons in female mice are resistant to chronic meth‐induced degeneration (Pilski and Graves 2023) and on data demonstrating no impact on SNc dopamine neuron mitochondrial density (Figure S1). However, despite these negative outcomes, mitochondrial function could be changed, and future studies are needed to fully understand the consequence of meth use in female subjects.

## 6 Conclusion

The present study provides evidence that chronic meth administration leads to an accumulation of dysfunctional mitochondria in the SNc. We found that chronic meth administration decreased the expression of fission and mitophagy‐associated proteins FIS1 and DRP1; this decreased protein expression was associated with increased mitochondrial density in SNc dopamine neurons, suggesting that chronic meth results in an accumulation of mitochondria. Despite this increase in mitochondrial density, high‐resolution respirometry revealed decreased complex I and complex I + II OXPHOS capacities and coupling efficiencies, suggesting mitochondria were functionally compromised after chronic meth administration. Importantly, the increase in mitochondrial density was prevented by MAO inhibition, linking meth‐induced mitochondrial accumulation to MAO‐dependent mitochondrial oxidative stress and SNc degeneration (Du et al. 2021; Graves et al. 2021, 2020; Pilski and Graves 2023). Taken together, the presented experimental outcomes provide evidence that the observed increase in mitochondrial density reflects an accumulation of dysfunctional mitochondria. The consequence of this would be a bioenergetic deficit that likely contributes to degeneration and increases the vulnerability of remaining SNc dopamine neurons to degeneration.

## Author Contributions

**Aishwarya Bhowmik:** formal analysis, methodology, investigation, writing – original draft, writing – review and editing. **Jennifer A. Wilking:** methodology, investigation, writing – review and editing. **Duha Alshareef:** methodology, investigation, formal analysis, writing – review and editing. **Colin Campbell:** methodology, writing – review and editing. **Steven M. Graves:** conceptualization, funding acquisition, methodology, writing – review and editing, writing – original draft.

## Funding

This work was supported by the National Institutes of Health (DA051450; SMG).

## Conflicts of Interest

The authors declare no conflicts of interest.

## Supporting information


**Figure S1:** Chronic meth administration had no impact on SNc dopamine neuron mitochondrial density in female mice.
**Figure S2:** Chronic meth administration increased mitochondrial DNA copy number in SNc.
**Table S1:** List of primers used to measure SNc mitochondrial DNA copy number.
**Table S2:** Shapiro–Wilk normality testing and outcomes.
**Table S3:** Reagents, Antibodies, and Instruments used.

## Data Availability

Data are available from the corresponding author upon reasonable request.
