# Supplementary material for: Chronic Methamphetamine Administration Increases Mitochondrial Density and Impairs Mitochondrial Function in the Substantia Nigra Pars Compacta
Source: J Neurochem. 2026 Sep 24;170(9):e70554. doi: 10.1111/jnc.70554 (PMC13613010; doi:10.1111/jnc.70554)
Supplement: Supplementary file 1 [file JNC-170-e70554-s001.pdf]

Chronic methamphetamine administration increases mitochondrial density and impairs mitochondrial function in the substantia nigra pars compacta

Aishwarya Bhowmik[1], Jennifer A. Wilking[1], Duha Alshareef[1], Colin Campbell[1], and Steven M. Graves[1]

[1]Department of Pharmacology, University of Minnesota, Minneapolis, MN 55455

Corresponding author:
Steven M. Graves, Ph.D.
Department of Pharmacology
University of Minnesota
3-106 Nils Hasselmo Hall
312 Church Street SE
Minneapolis, MN 55455
gravess@umn.edu
612-624-7335

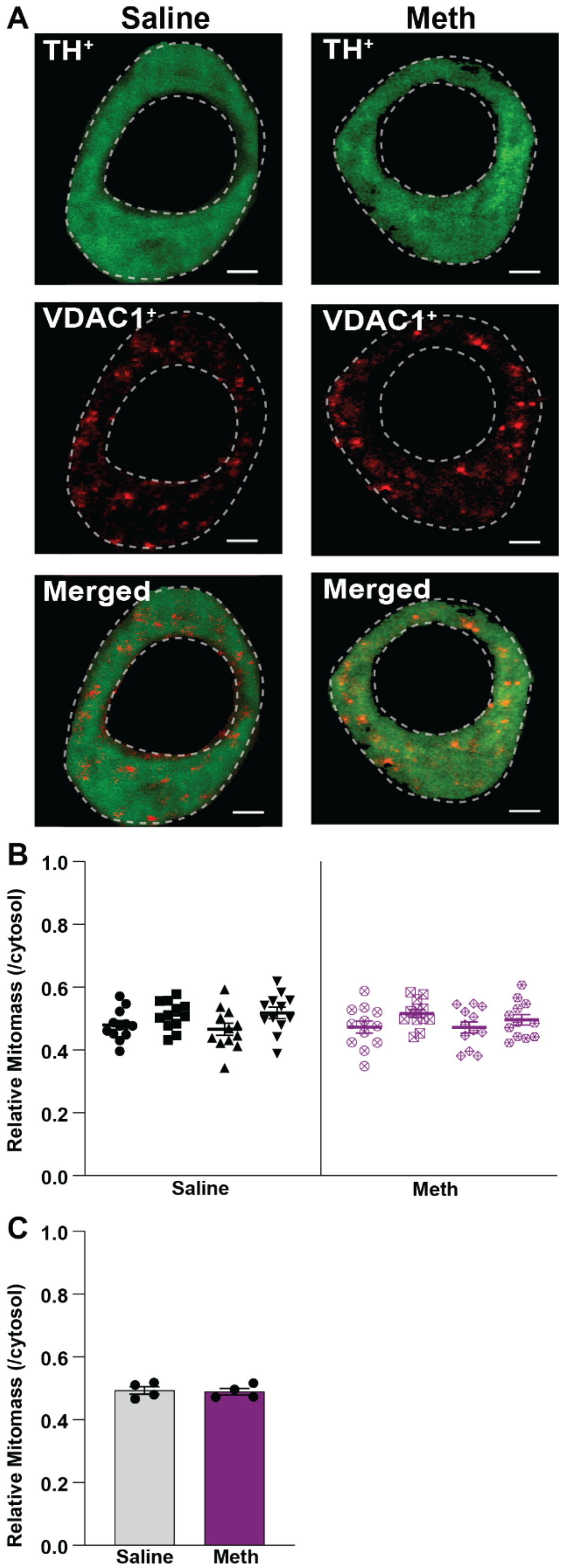

Supplementary Fig. 1. Chronic meth administration had no impact on SNc dopamine neuron mitochondrial density in female mice. Female mice were administered saline or meth for 28 days, after which mitochondrial density in SNc dopamine neurons was quantified. **(A)** Representative high-magnification images of SNc dopamine neurons showing tyrosine

hydroxylase staining (TH^+; green; top), mitochondrial labeling with voltage-dependent anion channel 1 ($VDAC1^+$; red; middle), and merged images (bottom) with a white dashed line marking the cell and nuclear membranes. Images were processed using Imaris MeasurementPro software, and the presented images are a single 2D plane from a 3D-reconstructed soma of female mice treated with saline or meth; scale bars denote 3 µm. **(B)** No differences were observed in the SNc dopamine neuron mitochondrial density between saline (n = 48 neurons/4 mice) and meth-treated (n = 48 neurons/4 mice) groups. Data analyzed using nested *t*-test (Saline: M = 0.4934, SD = 0.0586, Meth: M = 0.4892, SD = 0.0594, $t(6) = 0.2541$, $p = 0.8079$). Error bars indicate the mean ± standard error of the mean. **(C)** A secondary analysis was performed wherein neurons per mouse were averaged; results from the secondary analysis recapitulate findings using nested analyses. Data analyzed using unpaired *t*-test ($t(6) = 0.2539$, $p = 0.8081$, two-tailed). Histograms illustrate the mean ± standard error of the mean.

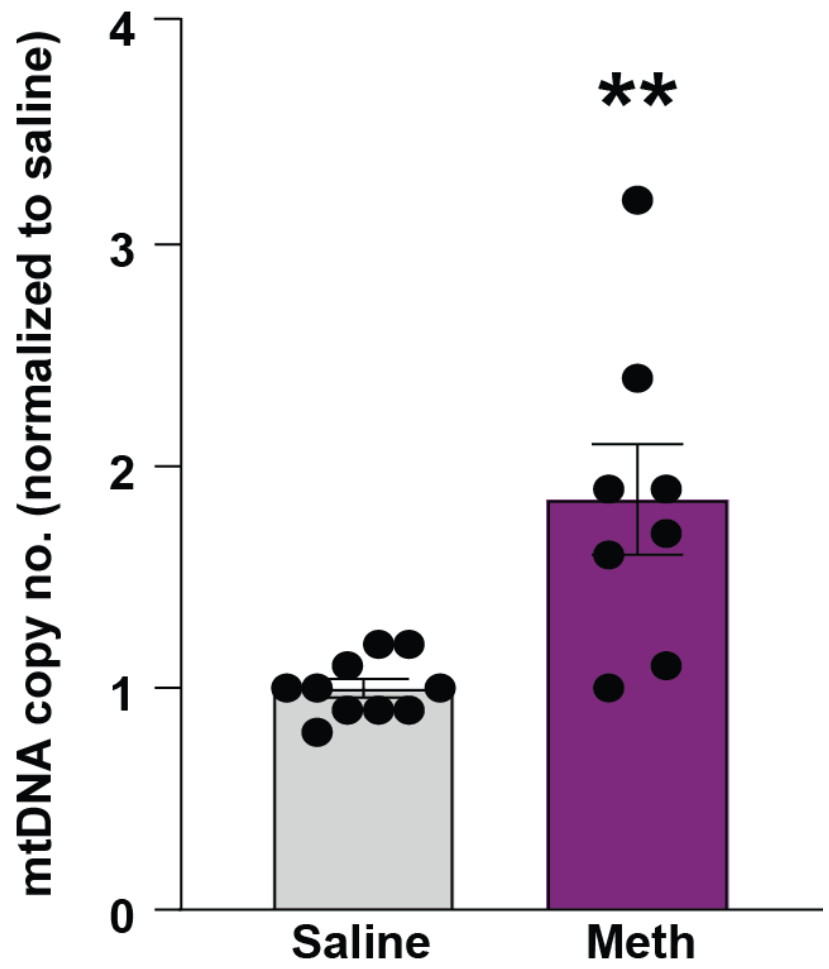

Supplementary Fig. 2. Chronic meth administration increased mitochondrial DNA copy number in SNc. Male mice were administered saline or meth for 28 days, after which mitochondrial DNA copy number in SNc was quantified. Mitochondrial DNA copy number in SNc increased in meth-treated mice (n = 8) compared to saline-treated mice (n = 10). Data analyzed using unpaired *t*-test ($t(16) = 3.747$, $^{**}p = 0.0018$, two-tailed). Histograms illustrate the mean ± standard error of the mean.

Supplementary Table 1. List of primers used to measure SNc mitochondrial DNA copy number.

Primer	Sequence
ND1 -F	CTAGCAGAAACAAACCGGGC
ND1 -R	CCGGCTGCGTATTCTACGTT
HK2 -F	GCCAGCCTCTCCTGATTTTAGTGT
HK2 -R	GGGAACACAAAAGACCTCTTCTGG

Supplementary Table 2. Shapiro-Wilk normality testing and outcomes.

FIGURES	SHAPIRO-WILK NORMALITY TEST	ANALYSIS	*p*-VALUE
**Figure 1B:** FIS1 Immunoblotting	Saline (W= 0.9169, *p*= 0.5104) and Meth (W= 0.9158, *p*= 0.5031) are normally distributed	Unpaired *t*-test	0.0006
**Figure 1D:** DRP1 Immunoblotting	Saline (W= 0.9211, *p*= 0.5370) and Meth (W= 0.9507, *p*= 0.7420) are normally distributed	Unpaired *t*-test	0.0034
**Figure 2E:** Cytosolic Volume	Saline (W= 0.9574, *p*= 0.7625) and Meth (W= 0.9587, *p*= 0.7708) are normally distributed	Unpaired *t*-test	0.3510
**Figure 3B:** Basal OCR	Saline (W= 0.9643, *p*= 0.8332) and Meth (W= 0.9301, *p*= 0.4485) are normally distributed	Unpaired *t*-test	0.4902
**Figure 3B:** G/M OCR	Saline (W= 0.9314, *p*= 0.4622) and Meth (W= 0.9154, *p*= 0.3204) are normally distributed	Unpaired *t*-test	0.2801
**Figure 3B:** P/M OCR	Saline (W= 0.9079, *p*= 0.2668) and Meth (W= 0.9507, *p*= 0.7420) are normally distributed	Unpaired *t*-test	0.0935
**Figure 3B:** ADP OCR	Saline (W= 0.9704, *p*= 0.8945) and Meth (W= 0.9113, *p*= 0.2898) are normally distributed	Unpaired *t*-test	0.0293
**Figure 3B:** Suc OCR	Saline (W= 0.9795, *p*= 0.9624) and Meth (W= 0.9241, *p*= 0.3927) are normally distributed	Unpaired *t*-test	0.0304
**Figure 3B:** CyC OCR	Saline (W= 0.9832, *p*= 0.9799) and Meth (W= 0.8858, *p*= 0.1518) are normally distributed	Unpaired *t*-test	0.0367
**Figure 3B:** Rot OCR	Saline (W= 0.9524, *p*= 0.6968) and Meth (W= 0.8833, *p*= 0.1425) are normally distributed	Unpaired *t*-test	0.0128
**Figure 3B:** AA OCR	Saline (W= 0.8934, *p*= 0.1849) and Meth (W= 0.9461, *p*= 0.6227) are normally distributed	Unpaired *t*-test	0.0695
**Figure 3B:** A/T OCR	Saline (W= 0.9276, *p*= 0.4586) and Meth (W= 0.9683, *p*= 0.8747) are normally distributed	Unpaired *t*-test	0.0032
**Figure 3C:** Complex I OXPHOS Capacity	Saline (W= 0.9605, *p*= 0.7921) and Meth (W= 0.9264, *p*= 0.4137) are normally distributed	Unpaired *t*-test	0.0287

**Figure 3C:** Complex I + II OXPHOS Capacity	Saline (W= 0.9732, *p*= 0.9192) and Meth (W= 0.9598, *p*= 0.7835) are normally distributed	Unpaired *t*-test	0.0345
**Figure 3C:** Complex I Coupling Efficiency	Saline (W= 0.9253, *p*= 0.4032) and Meth (W= 0.8599, *p*= 0.0762) are normally distributed	Unpaired *t*-test	<0.0001
**Figure 3C:** Complex I + II Coupling Efficiency	Saline (W= 0.9357, *p*= 0.5062) and Meth (W= 0.9091, *p*= 0.2752) are normally distributed	Unpaired *t*-test	<0.0001
**Figure 4B:** Basal H_2O_2 Production	Saline (W= 0.9601, *p*= 0.7866) and Meth (W= 0.8929, *p*= 0.1827) are normally distributed	Unpaired *t*-test	0.5852
**Figure 4B:** G/M H_2O_2 Production	Saline (W= 0.9313, *p*= 0.4608) and Meth (W= 0.9427, *p*= 0.5839) are normally distributed	Unpaired *t*-test	0.9502
**Figure 4B:** P/M H_2O_2 Production	Saline (W= 0.9726, *p*= 0.913) and Meth (W= 0.9629, *p*= 0.8188) are normally distributed	Unpaired *t*-test	0.2258
**Figure 4B:** ADP H_2O_2 Production	Saline (W= 0.8283, *p*= 0.0319) and Meth (W= 0.9062, *p*= 0.2557) are not normally distributed	Mann-Whitney test	0.3930
**Figure 4B:** Suc H_2O_2 Production	Saline (W= 0.9375, *p*= 0.5254) and Meth (W= 0.9287, *p*= 0.4354) are normally distributed	Unpaired *t*-test	0.8073
**Figure 4B:** CyC H_2O_2 Production	Saline (W= 0.9130, *p*= 0.3020) and Meth (W= 0.8691, *p*= 0.0976) are normally distributed	Unpaired *t*-test	0.9845
**Figure 4B:** Rot H_2O_2 Production	Saline (W= 0.9715, *p*= 0.9044) and Meth (W= 0.8130, *p*= 0.0209) are not normally distributed	Mann-Whitney test	0.7394
**Figure 4B:** AA H_2O_2 Production	Saline (W= 0.9345, *p*= 0.4933) and Meth (W= 0.7762, *p*= 0.0109) are not normally distributed	Mann-Whitney test	0.2110
**Figure 4B:** A/T H_2O_2 Production	Saline (W= 0.9076, *p*= 0.2647) and Meth (W= 0.9060, *p*= 0.2546) are normally distributed	Unpaired *t*-test	0.5580
**Supplementary Figure 1C:** Female Mitochondrial Density	Saline (W= 0.9087, *p*= 0.4755) and Meth (W= 0.8785, *p*= 0.3321) are normally distributed	Unpaired *t*-test	0.8081

**Supplementary Figure 2:** Mitochondrial DNA copy number	Saline (W= 0.9184, *p*= 0.3435) and Meth (W= 0.9321, *p*= 0.5350) are normally distributed	Unpaired *t*-test	0.0018

Supplementary Table 3. Reagents, Antibodies, and Instruments used.

REAGENT/ANTIBODY/INSTRUMENT	VENDOR	CATALOG NUMBER
Saline	General Laboratory Products	125EZ-7002-100
(+)-Methamphetamine hydrochloride	Sigma-Aldrich	M8750
Rasagiline mesylate	BioVision	2237-250
Isoflurane	Piramal Critical Care	66794-017
Phosphate-buffered saline	Sigma-Aldrich	P4417
Mouse brain matrix	ASI Instruments	RBM-2000C
Biopsy punch needle	Ted Pella	15115
1x RIPA buffer	Millipore Sigma	R0278
Protease inhibitor cocktail powder with EDTA	Sigma-Aldrich	P8340
Pierce BCA protein assay kit	Thermo Fisher Scientific	A65453
2X Laemmli Sample Buffer	Bio-Rad	1610737
β-mercaptoethanol	Bio-Rad	1610710
Precision plus protein all blue prestained protein standard	Bio-Rad	1610373
16.5% Mini-PROTEAN Tris/Tricine precast protein gel	Bio-Rad	4563063
12% Mini-PROTEAN TGX precast protein gel	Bio-Rad	4561045
0.45 μm nitrocellulose membrane	Bio-Rad	1620115
Fission 1 protein (FIS1)	GeneTex	GTX111010
β-Actin	Proteintech	66009-1
Dynamin-related protein 1 (DRP1)	Abcam	ab184247
Goat anti-rabbit Alexa Fluor 800 Plus	Invitrogen	A32735
Goat anti-mouse Alexa Fluor 680	Invitrogen	A21057
LI-COR Odyssey IR Imager	LICORbio	ODY1512
Ketamine	Dechra	07PG-KET50063-0518
Xylazine	Dechra	04SD-ROM21003-1121

Paraformaldehyde	Electron Microscopy Sciences	15735
Microtome	Leica	SM2010R
Tyrosine hydroxylase (TH)	Invitrogen	MA1-24654
Donkey anti-mouse IgG Alexa Fluor 488	Invitrogen	A21202
Voltage-dependent anion-selective channel protein 1 (VDAC1)	Abcam	ab15895
Donkey anti-rabbit IgG Alexa Fluor 555	Invitrogen	A31572
Formic acid	MilliporeSigma	695076
Normal donkey serum	MilliporeSigma	566460
Triton X-100	Sigma-Aldrich	T8787
Phosphate-buffered saline-TWEEN 20	Sigma-Aldrich	P3563
Donkey Anti-mouse IgG FAB fragment	JacksonImmunoResearch	715-007-003
Microscope glass slides with frosted end	Electron Microscopy Sciences	71870-01
ProLong Diamond Antifade Mountant	Invitrogen	P36970
Apotome fluorescence microscope	Zeiss	440762-9904-000
Qiagen DNeasy blood & tissue kit	Qiagen	69506
NanoDrop 2000 spectrophotometer	Thermo Scientific	ND-2000
1X SYBR Green	Applied Biosystems	43-444-63
QuantStudio 3 Real Time PCR System	Applied Biosystems	A28136
Oroboros NextGen-O2k Fluo system	Oroboros Instruments	10103-03
MES potassium salt (K-MES)	Sigma	M0895
Potassium chloride (KCl)	Sigma	P9541
Potassium phosphate dibasic (K_2HPO_4)	Sigma	P3786
Magnesium chloride hexahydrate ($MgCl_2.6H_2O$)	Sigma	M2670
Bovine serum albumin (BSA)	Sigma	A3803
Ethylene glycol-bis (2-aminoethylether)-N,N,N′,N′-tetraacetic acid (EGTA)	Sigma	E4378
Creatine monohydrate	Sigma	C3630
Amplex UltraRed (AmR)	Invitrogen	A36006

Horseradish peroxidase (HRP)	Sigma	P8375
Superoxide dismutase (SOD)	Sigma	S8160
Imidazole	Sigma	12399
Dithiothreitol (DTT)	Research Products International	C-1029
Taurine	Sigma	T8691
Adenosine 5′-triphosphate<br>Disodium salt hydrate (ATP)	Sigma	A6419
Phosphocreatine disodium salt hydrate	Sigma	P7936
Saponin	Sigma	S4521
L-Glutamic acid, sodium salt-hydrate (Glutamate)	Sigma	G5889
L-Malic acid (Malate)	Sigma	M7397
Sodium pyruvate	Sigma	P2256
Adenosine 5'-diphosphate<br>Mono potassium salt (ADP)	Sigma	A5285
Succinate	Sigma	S2378
Cytochrome C	Sigma	C2506
Rotenone	Sigma	R8875
Antimycin A	Sigma	A8674
Ascorbate sodium salt	Sigma	A7631
N,N,N′,N′-tetramethyl-p-phenylenediamine dihydrochloride	Sigma	T3134

Supplementary. Raw full Western blot image- FIS1

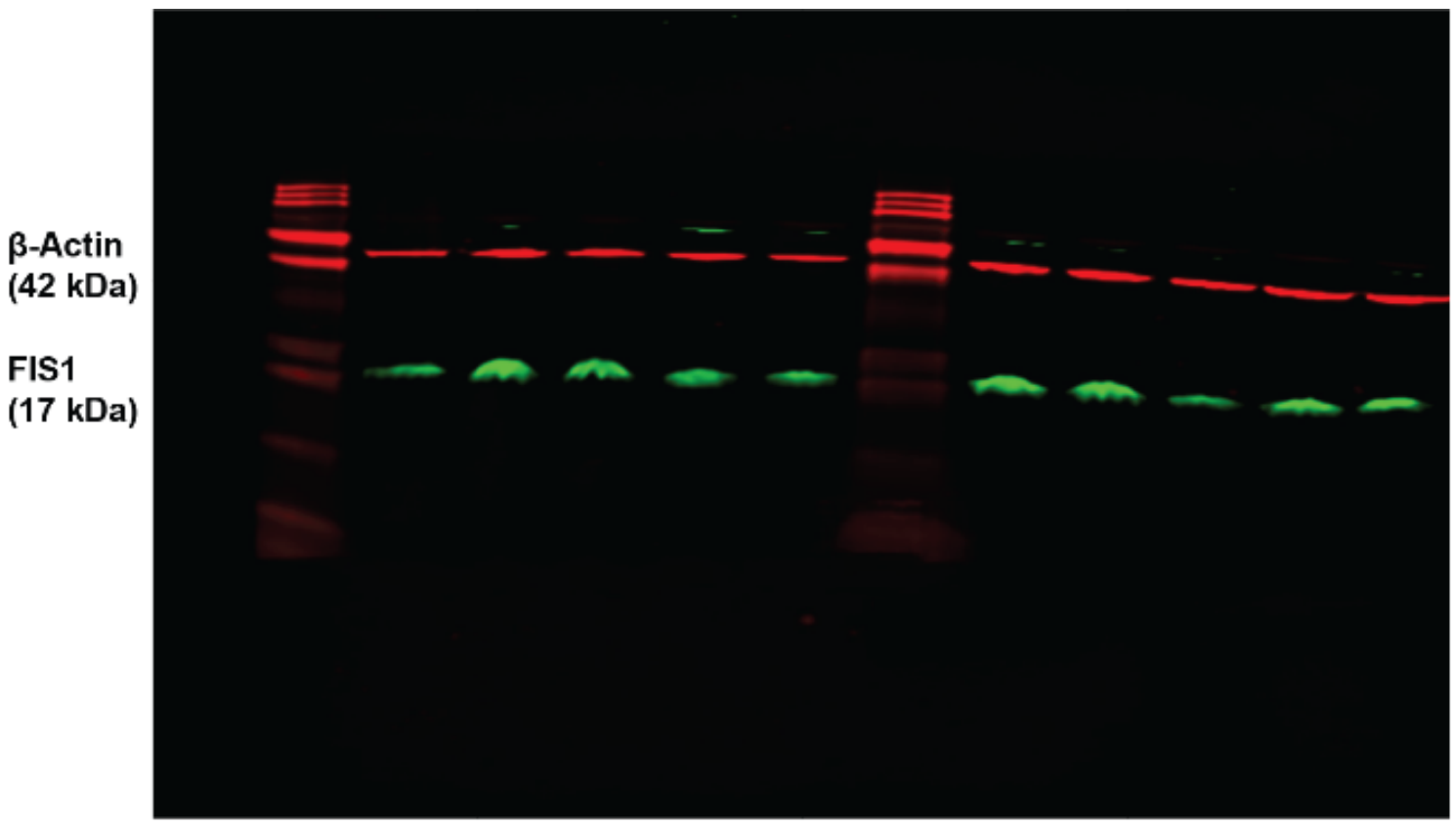

Saline vs. Chronic meth- FIS1 expression (unmodified)

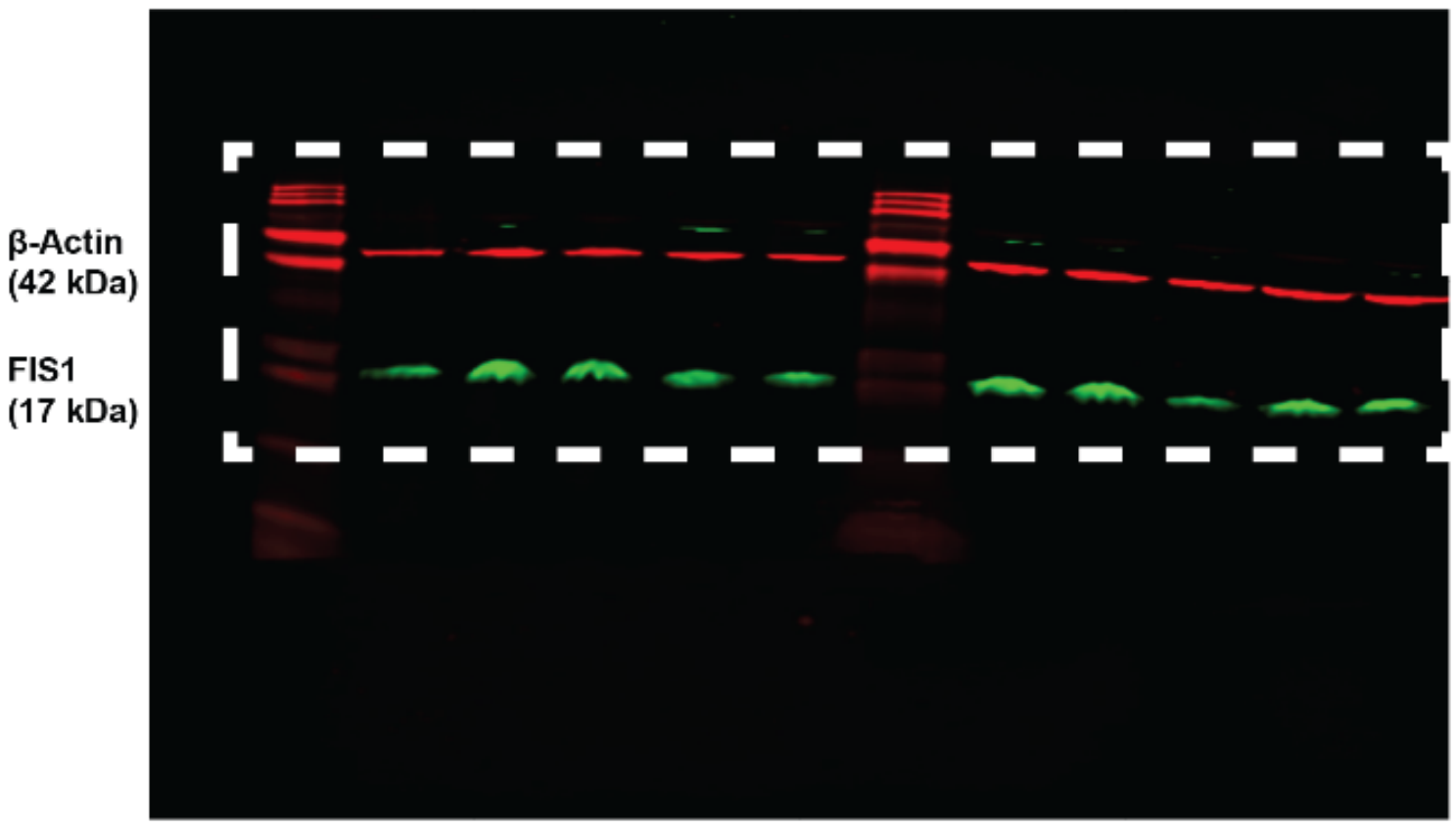

Saline vs. Chronic meth- FIS1 expression (unmodified- Boxed)

Supplementary. Raw full Western blot image- DRP1

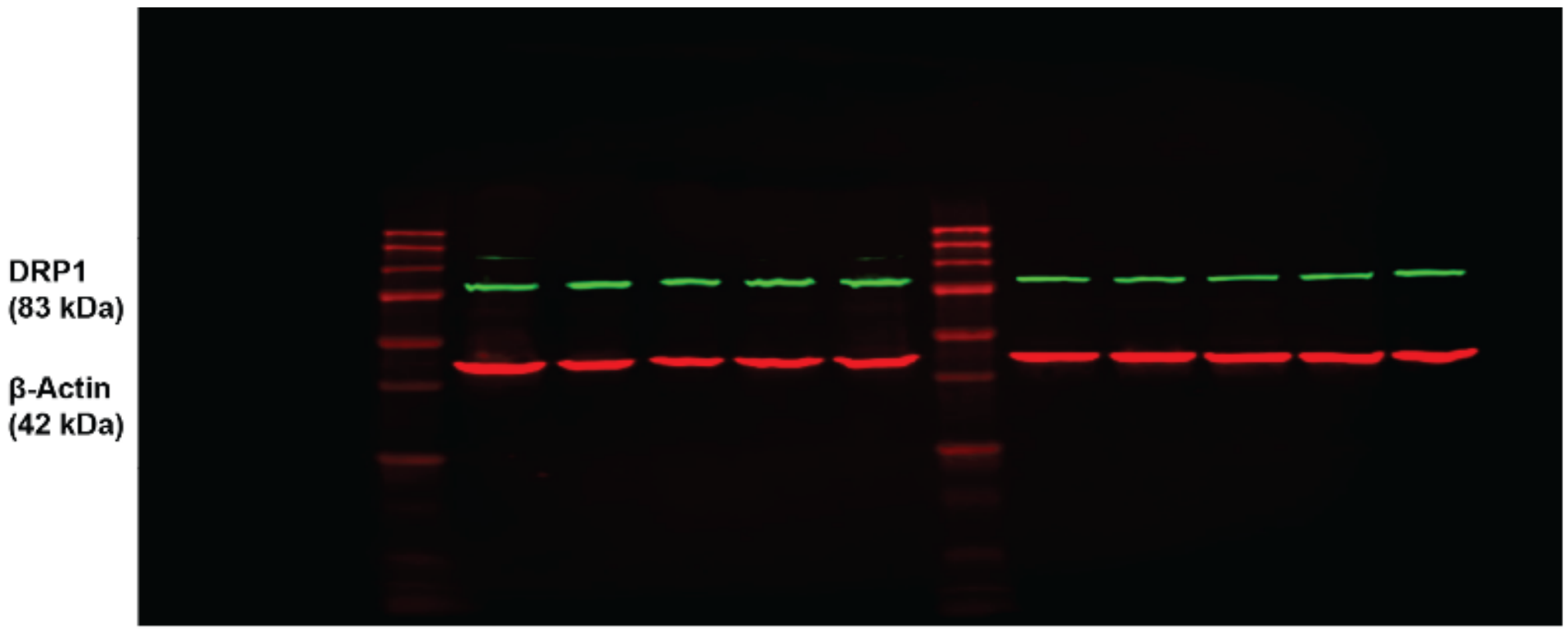

Saline vs. Chronic meth- DRP1 expression (unmodified)

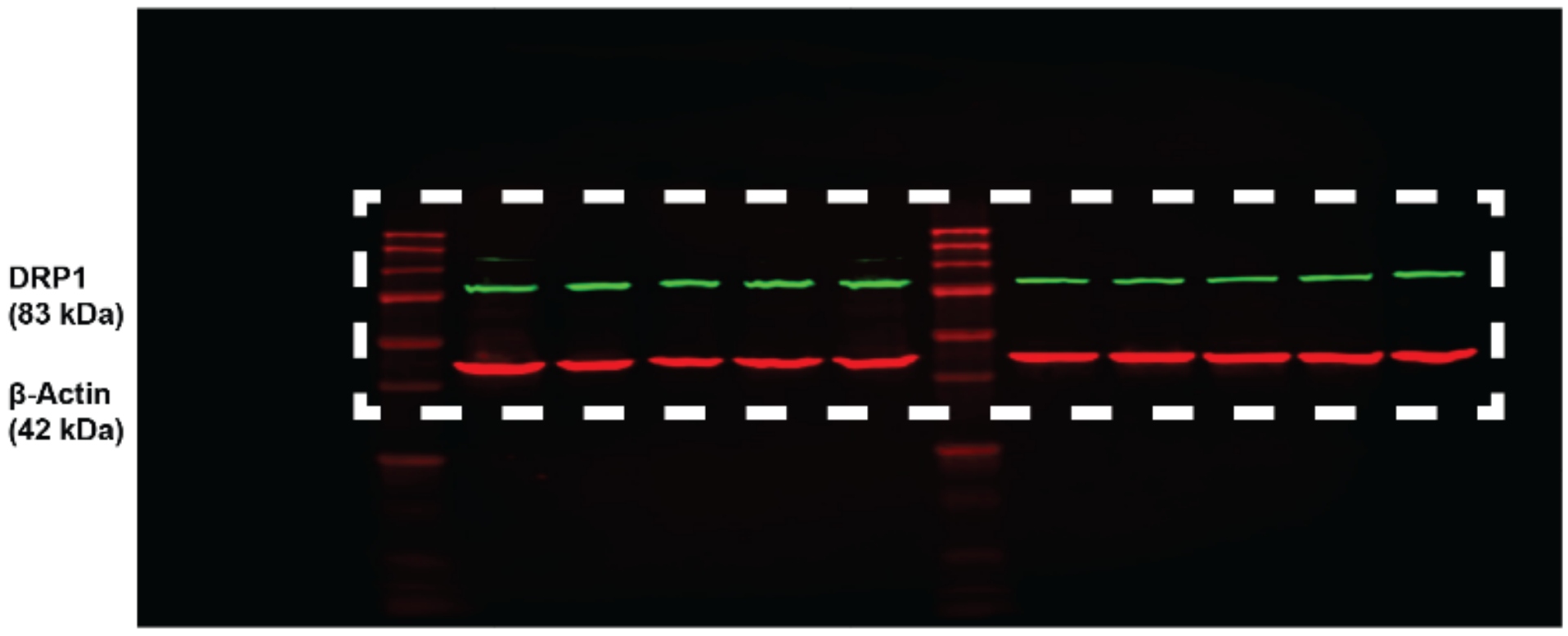

Saline vs. Chronic meth- DRP1 expression (unmodified- Boxed)
